# Editorial: Microbial Ecotoxicology

**DOI:** 10.3389/fmicb.2020.01342

**Published:** 2020-06-26

**Authors:** Stéphane Pesce, Jean-François Ghiglione, Edward Topp, Fabrice Martin-Laurent

**Affiliations:** ^1^INRAE, UR RiverLy, Villeurbanne, France; ^2^CNRS, UMR 7621 Laboratoire d'Océanographie Microbienne, Sorbonne Université, Observatoire Océanologique de Banyuls-sur-Mer, Banyuls-sur-Mer, France; ^3^Agriculture and Agri-Food Canada, University of Western Ontario, London, ON, Canada; ^4^AgroSup Dijon, INRAE, Univ. Bourgogne, Univ. Bourgogne Franche-Comté, Agroécologie, Dijon, France

**Keywords:** biodegradation, biodiversity, bioindicator, communities, ecosystem functions, pollutants, risk assessment

In the age of the Anthropocene, the world is facing unprecedented environmental challenges that have multifactorial and interlinked causes including population growth, pollution, and climate change. The “One Health” and “EcoHealth” paradigms emphasize the urgent need to protect ecosystem health in order to ensure human well-being and food security (Naeem et al., 2016; Destoumieux-Garzón et al., 2018). It is noteworthy that the majority of the 17 United Nations Sustainable Development Goals (UN-SDGs) fundamentally link environmental health to human health and well-being (Blicharska et al., 2019).

Within this context, mitigating anthropogenic impacts on ecosystem functions and services is a paramount challenge. Perhaps because they are not visible, microorganisms that provide or support key ecosystem services have generally been neglected as endpoints of concern in environmental risk assessment frameworks (Brandt et al., 2015). This, in spite of the fact that microbial communities deliver ecological processes and ecosystem services that are essential to life on earth (Cavicchioli et al., 2019). Exposure to inorganic or organic chemical pollutants, sometimes at very low concentrations, has the potential to kill or inhibit sensitive environmental microorganisms, or disrupt their activities. As with all biology on earth, microorganisms are subject to multiple physical and chemical stressors, including mixtures of commercial chemicals, pharmaceuticals, pesticides, and other agents that reach the environment either by design or in waste streams. Setting acceptable standards for chemical pollution on the basis of microbial impacts remains a significant challenge (Rockström et al., 2009).

Over the last few decades, a wide range of studies has investigated the interactions between microorganisms and pollutants at different biological scales ranging from the molecular to community levels. These studies have contributed to the emergence and the development of a new Research Topic designated “microbial ecotoxicology” (Ghiglione et al., 2016), built on key concepts from both microbial ecology and “classical” ecotoxicology. This Research Topic has recently benefited from tremendous technological improvements in several related fields, including environmental chemistry, microbiology, and microbial ecology as well as molecular and so called “omics ecology” (Marco and Abram, 2019). Indeed, a wide range of tools are now available to characterize microbial responses at different biological levels following exposure to a large variety of pollutants and their transformation products. Those concern so-called “legacy pollutants” (e.g., metals and metalloids, pesticides, chlorinated solvents or polycyclic aromatic hydrocarbons, PAHs), but also pollutants of emerging concern (e.g., pharmaceuticals, nanoparticles, plastic debris, biopesticides, or cyanotoxins). These responses are very complex and include reciprocal interactions because of the capacity of microorganisms to modify the bioavailability of pollutants and to transform or degrade many of them. Microbially biodegradable pollutants include substances that were initially specifically designed to inhibit microbial activities, such as some antibiotics (Cycon et al., 2019). Therefore, and as nicely illustrated in the present Research Topic bringing together 41 articles co-authored by 308 investigators, microbial ecotoxicology is a growing Research Topic that overcomes disciplinary approaches and compartment boundaries within ecosystems to assess the interactions between both prokaryotic and eukaryotic microorganisms and a wide range of pollutants with the final objective of tackling urgent environmental and societal challenges.

## Microbial Evolution and Adaptation to Pollutants: Resulting Environmental Benefits and Associated Ecological Costs

Mechanistic and evolutionary approaches are still needed to characterize and understand how microorganisms adapt to pollutants, not only to tolerate, resist or transform them, but also in some cases to take benefit from them as energy sources for metabolism and growth. Research in microbial ecotoxicology continuously contributes to identify and characterize undescribed mechanisms involved in detoxification and/or degradation of pollutants by different kinds of microorganisms. Bacterial membranes (Amin and Latif), surface layers (Chandramohan et al.), and external components such as extracellular polymeric substances constituting biofilm matrix (Mosharaf et al.) provide a barrier protecting microorganisms against diverse pollutants. This was nicely illustrated by Amin and Latif who coupled heteronuclear single-quantum coherence nuclear magnetic resonance (HSQC NMR) and scanning electron microscopy (SEM) to study and visualize the accumulation of mercury (Hg) on the cell surface of the Hg-resistant strain *Enterobacter* sp. AZ-15. Microbial detoxification systems involving pollutant sequestration are potentially useful to remove these substances from contaminated environments, such as wastewater (Mosharaf et al.). Extracellular enzymes can also be liberated in the surrounding environment to protect microorganisms through detoxification. Therefore, Karich et al. showed that two fungal unspecific peroxygenases from the basidiomycetous *Agrocybe aegerita* and *Marasmius rotula* were able to transform most of the compounds listed as priority pollutants by the United States Environmental Protection Agency (EPA). The fungal role in pollutant biodegradation was also highlighted by Carles et al. who described the cometabolic degradation of the herbicide nicosulfuron by *Plectosphaerella cucumerina AR1* isolated from *Alnus* leaf litter submerged in freshwater.

Evolution of microbial catabolic capabilities enhances the self-purifying capacity of contaminated environments, an important ecosystem service in both terrestrial and aquatic systems. However, studying and understanding the mechanisms that mediate biodegradation activity of recalcitrant pollutants remains necessary to better estimate the efficiency of such microbial processes and the resulting effects on pollutant fate and persistence in the different environmental compartments. For instance, Fanesi et al. demonstrated through the use of innovative vibrational spectroscopy techniques that soil content in organic matter and mineral particles is a strong driver of the metabolic profile of a PAH-degrading bacterium, *Pseudomonas putida* G7. Such knowledge is of particular interest to develop, improve, and implement innovative and successful bioremediation strategies for polluted soils and surface or ground waters (Donati et al., 2019; Hermon et al.; Laroche et al.; Spini et al.). As an example, a critical review of the role of marine microorganisms in the biodegradation of plastic debris and on the relevance of current standard tests for plastic biodegradability in seawater ecosystems is provided in the present Research Topic (Jacquin et al.). This review is complemented by an original study that described the successive phases of colonization, growing, and maturation phases of marine biofilms on non-biodegradable and biodegradable plastics (Dussud et al.).

Besides potential environmental benefits due to the development of biodegradation capacities, adaptation to pollutants is, on a one hand, a key process that confers resistance and resilience in exposed microbial communities supporting the ecological functioning of contaminated ecosystems (Pesce et al., 2017; Capdeville et al.). On the other hand, mechanisms and processes driving microbial adaptation to pollutants at individual level can represent a genetic burden decreasing the fitness of adapted populations when the selection pressure by the pollutant is released (Changey et al., 2011). Adaptative responses to pollutants, including notably the acquisition of tolerance, can be studied at the microbial community level. The emergence, at the end of the twentieth century, of the concept of pollution induced community tolerance (PICT; Blanck et al., 1988), is one of the best illustrations of this kind of approach, which is still currently used in microbial ecotoxicology to assess the causal relationship between pollutant exposure and microbial responses (e.g., Brandt et al., 2015; Tlili et al., 2016). However, there is evidence that PICT is generally associated with a decrease in diversity and the associated ecological functions (Mahamoud Ahmed et al., 2020) in line with the paradigm that biodiversity serves as ecological insurance (Yachi and Loreau, 1999). These resulting costs of tolerance can be viewed as ecological costs of adaptation (Clements and Rohr, 2009). Indeed, loss of diversity in microbial communities following pollutant exposure may not only alter the functions they support but also increase their vulnerability to further environmental disturbances. Conversely, environmental stressors (such as warming; Lambert et al., 2017 or hydrodynamics; Polst et al.) can influence microbial diversity and modify the capacity of microbial communities to tolerate pollutant toxicity. The development of pollutant-resistant species can also modify community functional pattern and alter some metabolic capacities (Cycon et al.). Moreover, within the One Health framework, the development or acquisition of resistance to antibiotics by environmental microorganisms is contributing to the emergence of multi-drug-resistant human and animal pathogens thus endangering livestock production and the efficacy of medicines to treat infections (Smalla et al., 2016; Topp et al., 2018).

## Pollutant Effects on Microbial Communities Threaten Ecosystem Biodiversity and Functions in All Environmental Compartments

Since the early 2000s, ecotoxicological studies (including “microbial ecotoxicology”) have progressively moved toward experimental designs that better assess and predict the ecological risks of a given chemical stressor under realistic environmental conditions. Several articles of this Research Topic highlight the potential effects of a wide range of pollutants on microbial structure, diversity and functions in contaminated soils (Borymski et al.; Cycon et al.; Mallet et al.; Simonin, Colman et al.; Simonin, Cantarel et al.), wastewaters (Guo et al.), continental (Chonova et al.; Freixa et al.; Pei et al.) and coastal surface water (Coclet et al.; Corcoll et al.; Zouch et al.), groundwater (Crampon et al.; Imfeld et al.), and sediments (Mahamoud Ahmed et al.). Such effects on microbial communities can threaten important ecosystem functions and services such as agricultural soil aggregation (Crouzet et al.), wastewater treatment (Guo et al.), primary production (Corcoll et al.), organic matter decomposition and nutrient cycling (Mahamoud Ahmed et al.; Rossi et al.; Simonin, Colman et al.), or energy transfer among trophic levels (Crenier et al.). Unsurprisingly, a large proportion of the above-mentioned studies concerns metal pollutants that are ubiquitously found in the environment due to the combination of natural and anthropogenic sources. Therefore, this Research Topic provides further evidence that metals are one of the major drivers of microbial community structure and diversity, affecting both bacteria, archaea and algae and the functions they ensure within ecosystems (Borymski et al.; Pei et al.; Coclet et al.; Corcoll et al.; Zouch et al.; Mahamoud Ahmed et al.). However, metals are also present in the environment as nanoparticles due to their high production and use for numerous applications. Simonin, Colman et al. and Simonin, Cantarel et al. showed that copper-based nanopesticides used as a fungicide and bactericide can impact microbial processes involved in soil carbon and phosphorus cycling after chronic exposure, revealing that short-term studies may underestimate the ecotoxicological risks of these new generation of metallic pesticides on soil microbial communities and ecosystem functioning. Other metal nanoparticles, such as zero-valent iron nanoparticles that are used for remediation of different kinds of pollutants (Crampon et al.) or silver nanoparticles that are used for antimicrobial treatment (Guo et al.), were also shown to present risks for bacterial community structure and metabolic potential. As nanopesticides, biobased pesticides are often viewed as a safe alternative to synthetic organic pesticides in agriculture to achieve the agroecology transition (Seiber et al., 2014). However, ecotoxicological risks and effects of these natural active substances are still under documented (Amichot et al., 2018). The study of Mallet et al. provides evidence that natural β-triketone herbicide leptospermone can impact the composition and diversity of fungal communities in arable soils. All these results underline the need of incorporating microbial endpoints for environmental risk assessment and homologation processes of novel agrochemicals such as nanopesticides or biobased pesticides. Those are viewed as pollutants of emerging concerns, such as pharmaceuticals or plastics, which are also taken into consideration in this Research Topic (Chonova et al.; Cycon et al.; Dussud et al.; Freixa et al.; Jacquin et al.; Orlewska et al.). Cyanotoxins are also sometimes cited as pollutants of emerging concerns (Snow et al., 2017) that can be harmful to both environmental and human health. Using a metabolomics approach, Le Manach et al. reported the production of a wide set of metabolites by 24 *Microcystis* strains, including different classes of cyanotoxins together with a large set of unknown molecules.

However, assessing and predicting the consequences of legacy pollutants or pollutants of emerging concerns on ecosystem functions and services remains a challenge because multiple stressors proceed simultaneously on complex interconnected biological communities. For a better understanding of the chain of causality starting from pollutant exposure to microbial community responses and then mediated ecosystem alteration, microbial ecotoxicological approaches and indicators that take into account the possible interactions between multiple pollutants are needed (Freixa et al.; Mahamoud Ahmed et al.). Therefore, Freixa et al. showed that carbon nanoparticles (fullerenes C_60_) can modulate the effects of organic pollutants on aquatic biofilm communities. However, they observed that both the magnitude and the direction of the interaction following joint exposure varied regarding the kind of organic pollutant, being sometimes antagonistic (e.g., fullerenes C_60_/diuron), synergistic (fullerenes C_60_/triclosan), or neutral (fullerenes C_60_/ venlafaxine) (Freixa et al.). Effect interactions can also vary according to the microbial endpoint considered. For instance, Mahamoud Ahmed et al. observed that arsenic and copper had an additive or synergetic negative impact on sediment denitrification while no interaction was recorded when considering other functions such as respiration or several extracellular enzymatic activities.

Moreover, interactions between pollutants and other environmental abiotic and biotic stressors must also be considered since they can modulate both the exposure and the sensitivity of microbial communities to toxicants. These parameters can be both physical, such as temperature (Pesce et al.) or hydrodynamic (Polst et al.), chemical, such as nutrients (Crenier et al.; Rossi et al.; Simonin, Cantarel et al.) or salinity (Rotini et al.), or biological (Bart et al.). More frequent studies evaluating the impact of combined or cumulative exposure to multiple chemical and non-chemical stressors on microbial communities in controlled conditions or along environmental gradients are being published (see above examples). Nevertheless, it is still difficult to go beyond these case-by-case studies and to generalize concepts that can be deployed widely in environmental risk assessment procedures. Large-scale initiatives (i.e., meta-studies) such as the “Microbiome Stress Project” proposed by Rocca et al. to leverage existing metagenomic studies assessing the response of microbial communities to various environmental stressors are timely. The crucial issue of data-sharing has recently been pinpointed by Eckert et al. (2020) who claimed that at least 20% of published metagenomic data are not generally accessible for other scientists, and lack robust metadata.

## Research in Microbial Ecotoxicology Should Improve Pollutant Risk Assessment and Ecosystem Quality Monitoring But Lacks Robust Transfer to Stakeholders and Environmental Managers

Microbial ecotoxicology is a growing Research Topic that contributes to improve knowledge on the microbial responses to pollutants under variable exposure scenarios. Furthermore, innovative methods and approaches are being developed including novel ecotoxicological endpoints (Sgier et al.), new types of bioassays (Morin et al.) and next generation methods, such as volatolomics to detect signatures of pollutant exposure (Hidalgo et al.). Efforts have also been made to develop and test bioindicator approaches to assess biological exposure to pollutants and/or resulting effects on ecological quality in contaminated ecosystems through the study of microbial communities naturally present in the different environmental compartments (Imfeld et al.; Pei et al.; Pesce et al., 2017). However, despite strong evidence of the relevance of developed methods and approaches, these remain case studies that do not entirely respond to the needs of environmental regulators and managers. Operational and generalizable procedures have not been examined so far, from sampling strategies to data analysis, including tests for robust interpretation methodologies with reference baselines and quality levels. Accordingly, changes in microbial assemblages are still not well taken into account for pollutant risk assessment and ecosystem quality monitoring, as regularly pointed out, for example, by the European Food Safety Authority (EFSA PPR Panel, 2013, 2017; EFSA Scientific Committee, 2016). As an example, potential impacts on microbial communities are completely ignored in the development of Environmental Quality Standards (EQS) in the EU water framework directive (WFD) that is the most significant European water legislation to date. These EQS mandate concentration thresholds that should not be exceeded for a range of selected pollutants to preserve ecological integrity in aquatic ecosystems. Only diatom biodiversity is used to assess the biological quality of water bodies within the WFD.

In 2016, Bengtsson-Palme and Larsson (2016) also underlined the need for considering the risks of antibiotic resistance development associated with antibiotic pollution and subsequent dissemination to human pathogens. They determined “predicted no effect concentrations” (PNECs) for 11 selected antibiotics based on available minimum inhibitory concentrations (MICs). Most of the PNECs were below available PNECs for ecotoxicological effects, suggesting that current environmental guidelines are not protective enough for environmental and human health (Tell et al., 2019).

## Key Challenges and Future Perspectives

Knowledge about the interactions between pollutants and microbial communities is continuously increasing. The following challenges, which are far from being exhaustive, underline the necessity (i) to reinforce disciplinary knowledge to better characterize microbial responses to pollutants at the species, population, and community levels but also (ii) to develop multi-disciplinary approaches combining among others microbial ecotoxicology, ecology, microbiology, toxicology, environmental chemistry and physics, big data science, modeling, as well as animal and human health sciences to better explore and predict the consequences of these responses on ecosystem and human health and well-being, in line with the “One Health” paradigm.

A major challenge is to go beyond the community level, which is commonly considered in microbial ecotoxicology (Ghiglione et al., 2016) in order to translate the microbial response to ecosystem scale. This is the policy-relevant scale to which prevention and mitigation strategies need to be deployed. This crucial and non-trivial issue goes beyond the sole scope of microbial ecotoxicology and needs the inclusion of the latest advances and concepts in microbial ecology (Graham et al., 2016) but also in general ecology to take into account the interactions between micro- and macro-organisms. The short- and long-term ecological costs of microbial adaptation processes (at both the species and community levels), as well as community and ecosystem resistance and resilience capacities that guarantee the preservation and/or the recovery of ecosystem biodiversity and functioning under pollutant exposure, need to be characterized. This cannot be done without taking into account the fact that microbial communities are generally chronically exposed to complex mixtures of pollutants and are otherwise subjected to a wide range of additional chemical, physical, and/or biological stressors. This is conceptually and technically challenging since the enormous diversity of pollutants and possible environmental stressors preclude the testing of every possible binary and multiple combinations. To face this gap, initiatives aiming to facilitate data sharing from multistress studies and their meta-analysis are greatly encouraged. Together with studies developing mechanistic models of interaction at different levels, these initiatives will be needed to produce robust and generalizable predictive models for the purposes of improving the *a priori* and *a posteriori* risk assessment of pollutants in the context of global change.

Another main challenge concerns the urgent need of extending our knowledge on the environmental factors (including pollutants) and the microbial mechanisms that contribute to the development, the proliferation and the dispersal of pathogenic microorganisms, microbial resistance to pharmaceuticals and microbial toxins in various environmental compartments. Managing microorganisms of human or animal health concern requires consideration of the environmental dimension of the One Health Continuum.

Finally, future research in microbial ecotoxicology should also gain in transferability to end-users, including policy-makers and environmental managers, to develop more effective policies and efficient regulations aiming at preserving our environment and health from the adverse effects of pollutants. We mentioned above the need of better considering microbial communities, their functional diversity and their role in the evolution and spreading of antibiotic-resistant pathogens in the elaboration of Environmental Quality Standards (EQS) and in homologation procedures (including those of nanopesticides or biobased pesticides). Given how rapidly research in microbial ecotoxicology and related methods are developing, it is a challenge to identify, standardize, and normalize the most relevant protocols for operational and regulatory applications. Indeed, standards are required (Philippot et al., 2012; Römbke et al., 2018) and large-scale studies are needed to perform meta-analyses and inform models to establish reference baselines, such as “normal operating ranges” of microbial functions (EFSA Scientific Committee, 2016). Moreover, one may not exclude the fact that the microbial world, which is most often invisible to the human eye, remains largely unknown to the general public and, even more worryingly, to a wide range of stakeholders and deciders. Among others, this is probably one of the main reasons why knowledge transfer from science to end-users and other stakeholders is particularly poor and has to be improved. It is thus important to have a dialogue between the research community, regulators, industry and broader society in order to share a common understanding making possible the achievement of a societal consensus defining the acceptable effect of pollutants on microbial communities and supported ecosystem services.

## Author Contributions

All authors listed have made a substantial, direct and intellectual contribution to the work, and approved it for publication.

## Conflict of Interest

The authors declare that the research was conducted in the absence of any commercial or financial relationships that could be construed as a potential conflict of interest.
